# The Lymphoscintigraphic Study of Unpredictable Head and Neck Cutaneous Melanoma Lymphatic Drainage

**DOI:** 10.3390/biomedicines8040070

**Published:** 2020-03-27

**Authors:** Valentina Lavelli, Cristina Ferrari, Giulia Santo, Corinna Altini, Andrea Ballini, Angela Sardaro, Margherita Fanelli, Antonio Rosario Pisani, Anna Giulia Nappi, Giuseppe Giudice, Giuseppe Rubini

**Affiliations:** 1 Nuclear Medicine Unit, Interdisciplinary Department of Medicine–University of Bari “Aldo Moro”, 70124 Bari, Italy; valentina.lavelli@gmail.com (V.L.); ferrari_cristina@inwind.it (C.F.); giuliasanto92@gmail.com (G.S.); corinna.altini@hotmail.it (C.A.); margherita.fanelli@uniba.it (M.F.); apisani71@libero.it (A.R.P.); anna.giulia.nappi@gmail.com (A.G.N.); Giuseppe.rubini@uniba.it (G.R.); 2 Department of Biosciences, Biotechnologies and Biopharmaceutics, Campus Universitario “Ernesto Quagliariello”, University of Bari “Aldo Moro”, 70125 Bari, Italy; andrea.ballini@uniba.it; 3 Department of Precision Medicine, University of Campania “Luigi Vanvitelli”, 81038 Naples, Italy; 4 Interdisciplinary Department of Medicine, section of Radiology and Radiation Oncology, University of Bari Aldo Moro, Piazza Giulio Cesare, 11–70124 Bari, Italy; 5 Division of Plastic and Reconstructive Surgery, Department of Emergency and Organ Transplantation, University of Bari, 70124 Bari, Italy; giuseppe.giudice@uniba.it

**Keywords:** Head and Neck Cutaneous Melanoma (HNCM), lymphatic drainage, Sidney Melanoma Unit Database, field cancerization

## Abstract

Head and neck cutaneous melanoma (HNCM) does not always follow standard lymphatic drainage; typical expected lymphatic pathways are associated with unexpected ones. The aim of this study was to investigate the relation between the primary HNCM sites and all possible lymphatic drainage pathways by lymphoscintigraphy with a special focus on the unexpected sentinel lymph node (SLNs) detection. We retrospectively analyzed 67 patients (46 M, 21 F; mean age 63 years) who underwent lymphoscintigraphy from January 2004 to November 2018. 99mTc-serum albumin was injected intra-dermally at the dose of 18–37 MBq in 0.2–0.4 mL. All patients underwent dynamic and static image acquisition. For all patients, the relation between the expected and unexpected SLNs was performed using the “Sidney Melanoma Unit Database” as our reference. The relation was performed also according to the primary HNCM localization. Cohens’ kappa was calculated. In 61/67 (91%) of patients, SLNs were detected only in predictable sites, while in six/67 (9%), unexpected SLNs were revealed. In all patients, the agreement proportion was 91% (95% confidence interval CI 0.8–0.96) and Cohen’s K was 0.11 (95% CI 0–0.43). Regarding the primary melanoma sites, the nasolabial field HNCM showed the highest rate of concordance (K = 0.60; 95%, CI 0.32–0.89) while the preauricular region HNCM revealed the highest rate of discordance with the clinically predictable drainage. The HNCM lymphatic drainage is extremely variable in regard to both the sites and the number of involved SLNs. The lymphoscintigraphic study is highly recommended to identify all possible SLNs in order to perform an accurate staging for all patients and to avoid missing unexpected SLNs.

## 1. Introduction

Head and neck cancer is a heterogeneous group of tumors and represents a challenge for diagnosis and treatment due to high morbidity and mortality. Melanomas represent only a small percentage of all head and neck cancers; 90% of malignant tumors are squamous cell carcinomas (SCCs), and the oral cavity represents the most affected area [1,2].

The GLOBOCAN 2018 estimated 354,864 oral cavity new cases, 287,723 melanoma new cases, 1.6% oral cavity deaths, and 0.6% melanoma deaths worldwide in 2018 [3].

In 2018 in Italy, the incidence was 13,700 new cases of skin melanoma. This represents the second most frequent type of cancer in men and the third in women under 50 years old [4]. Melanomas arising from sites in the head and neck accounted for approximately 20% of cases, which have been reported to have a worse prognosis compared with other sites [5].

The lymph node status remains the most important prognostic factor for recurrence in cutaneous melanomas, guiding treatment strategies [6]. Lymphoscintigraphy is used to study the lymphatic drainage and identify the sentinel lymph nodes (SLN), which are defined as any lymph node receiving (or the first lymph node to receive) direct lymphatic drainage from the primary tumor site [7].

A SLN biopsy can identify metastasis spread in lymph nodes. This procedure is currently used in melanoma staging; however, this is often difficult in head and neck cutaneous melanoma (HNCM), unlike other anatomical districts where lymphatic drainage is almost always clinically predictable.

Lymphatic drainage of HNCM and localization of SLNs is a challenge due to the complexity of this anatomical area, where more than 300 nodes are joined in an exceptionally compressed area [8]. Frequently, the lymphatic drainage of the head-neck district does not follow the standard drainage pathways; thus, it is essential to provide greater accuracy in identifying the transit nodes (i.e., those that lie between the primary tumor and a regional drainage basin and/or aberrant nodes (i.e., a nodal region outside the standard) [9,10,11].

The aim of this study was to investigate the relation between the primary HNCM sites and all the possible lymphatic drainage pathways by lymphoscintigraphy, with a special focus on the unexpected SLN detection.

## 2. Experimental Section

In our study, we retrospectively included 67 patients with head and neck melanoma who underwent lymphoscintigraphy from January 2004 to November 2018, followed by surgical excisions.

All patients had already given their consent for the use of their data for clinical research. Our institutional review board does not require ethical committee approval for the review of patients’ files. The Nuclear Medicine Unit, Interdisciplinary Department of Medicine–University of Bari “Aldo Moro” approved this study.

The study included 46 men (69%) and 21 women (31%). The mean age was 63 years (range 22–91). All patients presented a Breslow thickness of ≥0.5 mm and Clark level ≥ II. The radiotracer, 99mTc-serum albumin (Nanocoll^®^; GE Healthcare, Milan, Italy), was injected intradermally at a dose of 18–37 MBq in 0.2–0.4 mL; the dose was divided in two to four visible locations at the distance of 5 mm from the tumor margin or scar.

After the radiotracer injection, dynamic images were acquired with a GE Discovery 670 dual-headed camera (Discovery MN/CT, GE Healthcare, Haifa, Israel) using a specific protocol (energy window 140 keV ± 10%, low energy high resolution (LEHR) parallel-hole collimator, 20 frame/60 s for 20 min; matrix 128 × 128 size). At the end of the dynamic scintigraphy, static images were acquired.

The lymph node level system was defined from the standard nodes fields classification: occipital (including nuchal nodes), preauricular (including parotid nodes), postauricular, cervical levels I to V, submental, supraclavicular fossa, and axillary. These eleven theoretical levels were considered as possible drainage sites. In addition, the facial lymph node stations (buccinators, nasolabial, malar, and mandibular lymph node stations) were considered in our description.

The localization of the SLNs on lymphoscintigraphy were compared with the lymphatic drainage clinically predicted using the software created by the University of Auckland, based on the locations of the primary lesions [12,13].

First, we performed an analysis on the patients. Patients were considered with concordant results when the same lymphatic sites were found in both the Auckland database and our lymphoscintigraphy; patients were considered with no concordance results when our lymphoscintigraphy differed from the Auckland database in at least one of the lymph node levels. Cohen’s kappa was performed to evaluate the agreement.

In a second step, we performed an analysis on the sites. For each patient we considered 11 theoretical drainage levels. The sites were defined as concordant when the lymph node field was both present or both absent in the Auckland software and in our lymphoscintigraphy. The sites were defined as not concordant in the remaining cases. Cohen’s Kappa was performed to evaluate the agreement on the total of the hypothetical lymph node localizations. The same analysis was performed according to the primary localization.

## 3. Results

A total of 67 patients drained 127 lymph nodes. In 4/67 patients, four SLNs were detected for each patient. In 11/67, three SLNs were detected; in 26/67, two SLNs were detected; and in 26/67, one SLN for each patient was detected.

The mean number of SLNs per patient was 1.4. There were 61/67 patients (91%) who drained unilaterally. Bilateral drainage occurred in 6/67 patients (9%) and four of them drained from the scalp. One patient with posterior neck melanoma drained outside the neck to the bilateral axillae. There were 23/67 patients (34%) who drained in more than one lymph node of the same lymphatic drainage. In particular, 18 patients drained more than one SLN to the lateral cervical region.

**Figure 1:**
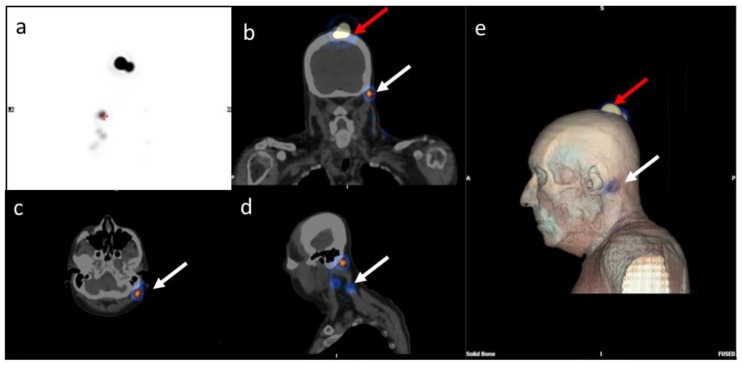
99mTc-serum albumin lymphoscintigraphy in a 65 year-old patient with scalp primary infiltrative head and neck cutaneous melanoma (red arrows). (**a**) maximum intensity projection (MIP), (**b**) coronal, (**c**) trans-axial, and (**d**) sagittal fused images, single-photon emission computed tomography/computed tomography (SPECT/CT). (**e**) A 3D reconstruction shows the sentinel lymph nodes drained to the preauricular and cervical level II-IV. Three sentinel lymph node (SLNs) were detected. (white arrows).

Figure 1 shows the lymphoscintigraphy of a 65-year-old man with melanoma of the scalp: three sentinel lymph nodes were detected in the lateral cervical levels (level II–V).

**Figure 2:**
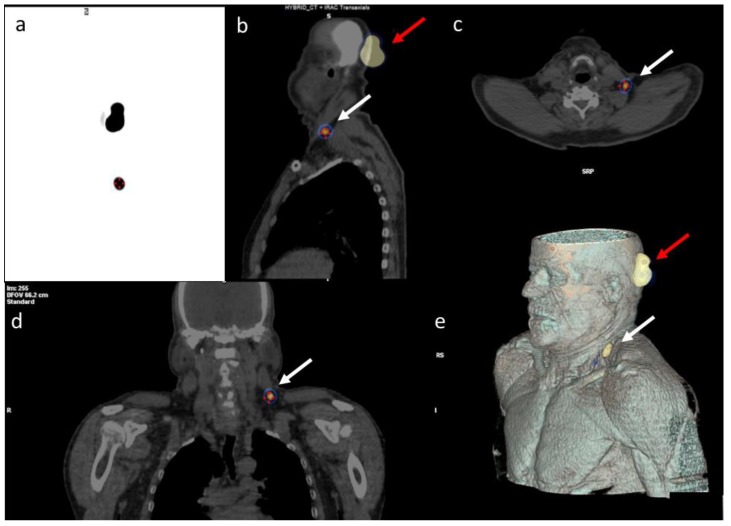
99mTc-serum albumin lymphoscintigraphy in a 64 year-old patient with a temporal region primary head and neck cutaneous melanoma. Breslow 2.5 mm. (red arrows). (**a**) MIP, (**b**) sagittal, (**c**) trans-axial, and (**d**) coronal fused images SPECT/CT. (**e**) A 3D reconstruction shows the sentinel lymph nodes drained to the supraclavicular region (white arrows).

Figure 2 shows a patient with melanoma of the temporal region; one unexpected lymph node was detected in the supraclavicular region.

Patients with melanoma of the scalp, ear, and preauricular region showed the highest number of sentinel lymph nodes (respectively, 21%; 18%; and 11%).

The number of patients for each primary melanoma site is reported in Table 1.

In 61/67 patients, the SLNs were found in the database of predicted drainage sites, whereas in 6/67 patients, we found unexpected drainage sites. Three out of six patients with a preauricular primary melanoma had an unexpected lymphatic drainage. One patient drained in a supraclavicular SLN, another patient in a submandibular one, and the third had two unexpected SLNs, both in the submandibular region.

The remaining three unexpected sentinel lymph nodes were all localized in the supraclavicular region, draining from the cervical, retroauricular, and nasolabial field primary sites. In all patients, the agreement proportion was 91% (95% confidence interval CI 0.8–0.96) but Cohen’s K was 0.11 (95% CI 0–0.43), so no concordance was found in the lymph node field clinically predicted by the Auckland software and our lymphoscintigraphy.

The distribution of the drainage sites of the 127 SLNs are described in Table 2.

Considering, for each patient, 11 hypothetical lymphatic levels, this resulted in 457 lymph nodes sites absent and 82 sites present with both Auckland’s Software and our lymphoscintigraphy; 182 were discordant results (K = 0.326; 95% CI, 0.26–0.39). The same analysis was performed stratifying for the primary site of melanoma and the results are reported in Table 3.

In the statistical analysis for the primary site of melanoma, two localizations deserved further considerations. For the primary melanoma of the nasolabial field, the concordance was moderate with a Cohen’s K of 0.60 (95% CI 0.32–0.89). On the contrary, the primary melanoma of the preauricular region showed no concordance with the clinically predictable drainage. Cohen’s K was 0.31 (95% CI 0.09–0.54). Overall, in this region, there was the highest rate of discordance with 3/7 (43%) of patients presenting unexpected lymphatic drainage. In particular, one of these patients presented only an unexpected lymphatic pathway in the submandibular region.

## 4. Discussion

Head and neck cancer represents a challenge due to the heterogeneous histological pattern of tumors in this anatomical area. Despite their different origins, etiopathogenesis, risk factors, and therapeutic and diagnostic strategies, the complex anatomy of the head and neck requires considerations of the important aesthetic and functional outcomes [14].

The most common head and neck cancer is squamous cell carcinoma (90%) [1], followed by other histological types, such as head and neck melanoma. Over the last half-century, the incidence of cutaneous melanoma has dramatically increased. One-third of all melanomas originate from the head and neck region, due to the constant sun exposure [15,16,17,18].

The treatment of malignant HNCM follows the same guidelines of melanomas of other sites of the body. The lymph nodal staging of the disease represents an important step to make decisions regarding adjuvant chemotherapy. The standard treatment is the complete wide local excision of the primary tumor followed by margins excisions. After excision of the primary tumor, lymphadenectomy of the lymph nodes involved is performed. For patients with positive lymphadenopathy by physical examination or imaging, a neck dissection is almost always performed. In the management of the N0, it is important to identify the lymphatic drainage of the primary tumor to evaluate if there may be an occult spread of melanoma to the regional lymph nodes and, thus, proceed to the sentinel lymph node biopsy (SNLB) [14].

A sentinel lymph node biopsy (SNLB) is recommended in patients with an intermediate thickness melanoma (Breslow thickness from 1.0 to 4.0 mm, T2 or T3). A SNLB can also be recommended in patients with a melanoma of greater thickness (Breslow > 4.0 mm, T4) [19]. The latest National Comprehensive Cancer Network (NCCN) guidelines of 2019 also suggested an SLN biopsy in patients with early stage (T1 or T2) oral cavity carcinoma as an alternative to elective neck dissection for identifying occult cervical metastasis. This could result in a reduction of morbidity and an improvement of the esthetic outcome. Patients with a positive SNLB must undergo complete neck dissection [20].

The identification and removal of sentinel lymph nodes in the head and neck can be difficult due both to the high anatomical complexity of this site, where numerous organs are present, and to the presence of more than 300 lymph nodes in the head and neck region [5].

As early as 1992, Morton et al. studied the correlation between each cutaneous region and a well-defined lymphatic drainage pathway. This drainage always occurs towards the sentinel lymph node [7]. The classic lymphatic drainage of cutaneous head and neck melanomas was described by O’Brien et al. based on a consecutive series of 183 neck dissection specimens and this model has been widely used to guide neck dissections. The same model was proposed to determine predictable lymphatic pathways, in particular, to differentiate an anterior lymphatic drainage from a posterior one [21].

The study of the possible lymphatic drainage has led to differentiate surgical strategies based on the site of the primary melanoma. For example, Goepfert et al. demonstrated that patients with a neck or scalp primary melanoma, localized posterior to a vertical line through the external auditory canal, required a postero-lateral neck dissection that included level II–V of the neck plus the retroauricular and suboccipital lymph nodes [22]. Patients with a scalp or neck primary melanoma, localized anterior to that vertical line, undergo a lateral neck dissection (levels II-IV) and a parotidectomy. Melanoma primary lesions on the face are treated with a supraomohyoid neck dissection (levels I–III). The parotid is a frequent site of metastasis from the temple, peri-auricular, and anterior scalp areas. For very anterior lesions occurring on the central face, chin, and neck, the parotid lymph nodes are not likely to be a site of melanoma metastasis [23,24].

The lymphatic patterns found by lymphoscintigraphy in the head and neck melanomas largely agreed with the anatomical model put by O’Brien et al.; however, some studies have evaluated that, in 8%–43% of patients, lymphoscintigraphy can point out unexpected drainage patterns [25,26].

This study was carried out to demonstrate the correlation between the localization of the primary tumor and lymphatic drainage with a special focus on unexpected SLN detection. We compared our lymphoscintigraphy results with those found on a digital software designed by the University of Auckland (New Zealand) based on a database that included 929 patients with HNCM, created by the Melanoma Institute Australia. A 3D computer model of lymphatic drainage of the skin was realized using melanoma lymphoscintigraphy [27].

The software shows the most likely lymphatic drainage field based on their sample according to the localization of the primary areas [12,13]. This software is available at http://sites.bioeng.auckland.ac.nz/hrey004/head/index.html.

The concordance was calculated first in all patients and, a second time, for each primary site of melanoma. No concordance was found between the localization predicted by the database, taken as a reference, and the lymphatic drainage sites found in our patients. We found unexpected lymphatic drainage in six patients (9%). This percent is in line with the current literature. Considering, for each patient, 11 hypothetical drainage levels, this resulted in 457 lymph nodes sites absent and 82 sites present with both Auckland’s Software and our lymphoscintigraphy; 182 were discordant results (K = 0.326; 95% CI, 0.26–0.39).

The preauricular region of primary melanomas presented the highest rate of discordance: 3/7 (43%) patients presented unexpected lymphatic drainage. One of these patients had only an unexpected lymphatic pathway in the submandibular level. The other two patients drained to the supraclavicular and submandibular regions. The remaining three unexpected lymph nodes were all localized in the supraclavicular level drained from the cervical, retroauricular, and nasolabial field melanoma primary sites.

In conclusion, 4/7 (57%) of the unexpected lymph nodes were supraclavicular. Therefore, we highly recommend nuclear medicine through lymphoscintigraphy in the head and neck cutaneous melanoma where lymphatic drainage pathways cannot be clinically predicted. Currently, the increased use of single-photon emission computed tomography/computed tomography (SPECT/CT) in addiction to planar lymphoscintigraphy allows for a better detection rate in the identification of SLN in melanoma patients, particularly in HNCM. Trinh et al. demonstrated how the SPECT/CT is able to better identify the supraclavicular localization of the SLNs and define the surgical strategy [28].

SPECT/CT becomes particularly important in the case of unexpected lymph nodes, as it allows for the study of not only the lymph node drainage pathway but also the anatomical region, allowing a clear preoperative evaluation.

## 5. Conclusions

The HNCM lymphatic drainage is extremely variable in regard both to the sites and number of involved SLNs. Our study demonstrated that it is not possible to clinically predict the lymphatic drainage of HNCM, and we highly recommend lymphoscintigraphic studies to identify all possible SLNs in order to perform an accurate staging for all patients and to avoid missing unexpected SLNs. This is important to avoid inappropriate or excessive dissections that could result in a functional or aesthetic limitation for patients. For these reasons, multidisciplinary management is essential for patients with head and neck cutaneous melanomas.

## Figures and Tables

**Table 1:** Number of patients for each primary melanoma site.

Primary Melanoma Site	Number of Patients
Scalp	14
Ear	13
Preauricular	7
Temporal	6
Cervical	4
Cheek	3
Retroauricular	3
Nasolabial fold	5
Nose	3
Neck	2
Front	2
Cervical (posterior)	1
Parietal/occipital	1
Subfrontal	1
Lower eyelid	1
Zygomatic	1

**Table 2:** Lymph node levels and the total number of sentinel lymph nodes per level.

SLN Levels	Number of SLNs
Cervical levels (II–V)	57
Preauricular	23
Cervical level I (or submandibular)	18
Postauricular	9
Occipital (or nuchal)	8
Supraclavicular	7
Axillar	2
Nasolabial	2
Submental	1

**Table 3:** Cohen’s K calculated for each primary melanoma site.

Primary	K	95% CI
Cervical	0.12	0‒0.30
Cervical (posterior)	0.56	0.06‒0.56
Cheek	0.31	0.09‒0.54
Ear	0.52	0.20‒0.84
Front	0.56	0.41‒0.71
Lower eyelid	0.62	0‒1
Nasolabial/fold	0.60	0.32‒0.89
Neck	0.30	0‒0.64
Nose	0.26	0.03‒0.48
Parietal/occipital	0.42	0‒0.88
Preauricular	0.31	0.09‒0.54
Retroauricular	0.24	0‒0.63
Scalp	0.25	0.15‒0.36
Subfrontal	0.42	0‒1
Temporal	0.34	0.14‒0.55
Zygomatic	0.61	0‒1
